# Study protocol for Norwegian Psychomotor Physiotherapy versus Cognitive Patient Education in combination with active individualized physiotherapy in patients with long-lasting musculoskeletal pain – a randomized controlled trial

**DOI:** 10.1186/s12891-016-1159-8

**Published:** 2016-08-05

**Authors:** Tove Dragesund, Alice Kvåle

**Affiliations:** 1 Department of Global Public Health and Primary Care, University of Bergen, Bergen, Norway; 2 Department of Occupational therapy, Physiotherapy and Radiography, Faculty of Health and Social Sciences, Bergen University College, Bergen, Norway

## Abstract

**Background:**

Norwegian Psychomotor Physiotherapy (NPMP) has been an established treatment approach for more than 50 years, although mostly in the Scandinavian countries, and is usually applied to patients with widespread and long-lasting musculoskeletal pain and/or psychosomatic disorders. Few studies have been investigating outcome of NPMP and no randomized clinical trials (RCT) have been systematically tried out on individuals.

**Methods/design:**

This is a study protocol for a pragmatic, single blinded RCT, which will take place in a city of Norway. The participants will be block randomized either to receive NPMP or Cognitive Patient Education in combination with active individualized physiotherapy (COPE-PT). The intervention will reflect usual care and will be conducted in physiotherapy clinics by five experienced physiotherapists in each of the two treatment approaches.

**Discussion:**

The findings of the present study may give an important contribution to our knowledge of the outcome of NPMP, on patients with long-lasting widespread musculoskeletal pain and/or pain located to the neck and shoulder region.

**Trial registration:**

The study has been registered with ClinicalTrials.gov (June 9 th 2015, NCT02482792).

**Electronic supplementary material:**

The online version of this article (doi:10.1186/s12891-016-1159-8) contains supplementary material, which is available to authorized users.

## Background

Musculoskeletal pain affects about three in four of the adult population in Norway during 1 month, and is most common among women, increase with age and prevalence seems to be stable over time [1, 2]. Long-lasting-, as well as widespread musculoskeletal pain also seem to be common [1, 3]. Musculoskeletal pain comprises nearly 40 % of sick leave in Norway [4] and 32 % of the disability pensions [5]. Because of the complexity and multi-factorial etiology of long-lasting musculoskeletal pain, targeted treatment is challenging and time consuming with a need of including physical, psychological and social aspects in the intervention [6, 7].

Norwegian Psychomotor Physiotherapy (NPMP) has been an established treatment approach for more than 50 years, although mostly in the Scandinavian countries. NPMP is usually applied to patients with widespread and long-lasting musculoskeletal pain and/or psychosomatic disorders. The perspective in NPMP is that physical, psychological and social strains may influence the whole body and can affect muscle tension, breathing, posture, balance, movements and flexibility. These elements are addressed when grasping the patient’s history of complaints, as well as during body examination and treatment [8]. The majority of patients referred to NPMP are women, their health problems have often lasted for several years and the treatment might be long-lasting because of the complexity of the pain disorder [9, 10].

Most studies of NPMP have focused on descriptions of different aspects in the treatment process, both from the patient’s and the physiotherapist’s perspective [11–16].

There are few studies investigating the outcome of NPMP treatment. However, Aabakken et al. [17] did a one-group prospective study of 152 patients with chronic pain receiving NPMP. After two and a half years, 72 % of the patients had achieved significant improvement regarding pain symptoms and everyday coping. In another prospective study [18] 60 patients with long-lasting musculoskeletal pain were included; 40 received NPMP and 20 were on a waiting- list for such treatment. After 12 months the 40 patients experienced reduced depression, anxiety, insomnia, fatigue and improved quality of life, while the patients on a 6 months waiting list had not changed. Only one RCT of NPMP has been performed, although only for groups and not on individuals, following a multi-model treatment program for patients with long-lasting musculoskeletal pain [19]. The study indicated that the patients receiving NPMP group treatment achieved fewer tender points, reduced distribution of pain and a higher rate of return to work after 1 year, compared to a control group of patients receiving usual follow-up at an out-patient rehabilitation clinic. However, the drop-out in the treatment group was large, making the results questionable. Another one-group prospective observational study of patients with low back pain receiving NPMP, showed that nine of the 12 included patients improved significantly regarding pain, flexibility and ability to relax [20].

In summary, few studies have been investigating outcome of NPMP and no RCT of the treatment approach have been systematically tried out on individuals.

### Purpose and research question

The main aim of the present study is to investigate the effectiveness of Norwegian Psychomotor Physiotherapy (NPMP) on pain, physical function, mental health, quality of life and sick leave in employees with long-lasting musculoskeletal pain or pain located to the neck and shoulder region, compared to employees receiving Cognitive Patient Education in combination with active individualized physiotherapy (COPE-PT).

The following research questions are posed:

- Is there a difference in pain intensity between workers who have received Norwegian Psychomotor Physiotherapy (NPMP) compared to those receiving Cognitive Patient Education and active, individualized physiotherapy (COPE-PT) at 3, 6 and 12 months after inclusion in the study?

Furthermore, do workers receiving NPMP improve more in function, mental health and quality of life, compared to those receiving COPE-PT at 3, 6 and 12 months after inclusion in the study?

- Is there a difference in sick leave 12 months after inclusion in the study between workers receiving NPMP, compared to the sick-listed COPE-PT participants?
- What characterizes those who are still sick-listed at 12 months compared to those who have returned to work? Is there an association between the tested function and self-reported measures on health and working ability?

## Methods and design

### Design and settings

**Fig. 1:**
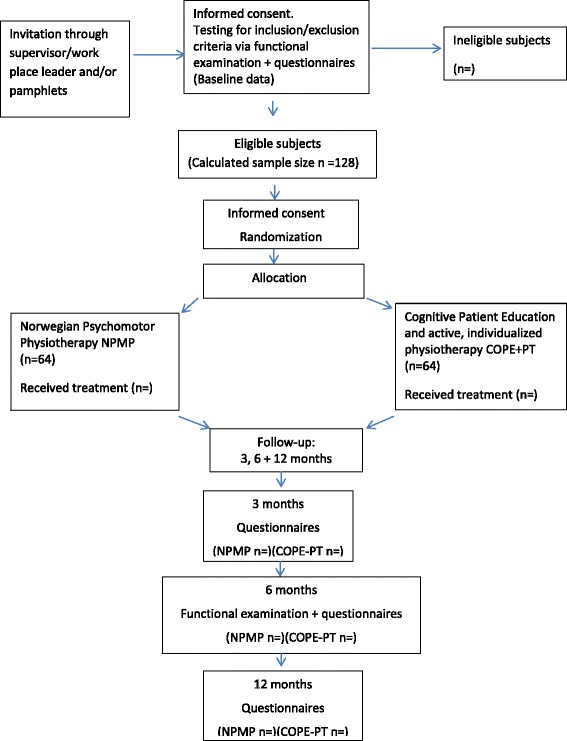
Flow diagram of the study protocol

This protocol describes a pragmatic single blinded randomised controlled study that will take place in a city of Norway. The study will follow the CONSORT 2010 checklist. The participants will be randomized either to receive Norwegian Psychomotor Physiotherapy (NPMP) or Cognitive Patient Education in combination with active, individualized physiotherapy (COPE- PT). In order to reflect usual care, the intervention will be pragmatic and conducted in physiotherapy clinics by five experienced physiotherapists in each of the two treatment approaches (Fig. 1). The study has been registered with ClinicalTrials.gov (June 2015, NCT02482792).

### Participants

Employees working in the Municipality of Bergen with long-lasting widespread musculoskeletal pain or pain in the neck and shoulders, are invited to participate in the study. They can have had several short sick-leaves during the last 2 years, or been sick-listed fulltime for < 6 months, or still be working despite their pain. The employees will get information about the project through their supervisors/work place leaders and/or through pamphlets at work. It is voluntary to participate and those who are interested will call for an appointment for an evaluation. On the first visit demographic data is collected and the participants will fill in questionnaires and undergo an examination of physical function (Tables 1 and 2). Those who fulfill the inclusion criteria will be invited to participate in the RCT (Fig. 1). Those not fulfilling the criteria (too little pain and functional problems) will get individual feedback and advice. Informed consent is first signed before the functional testing starts and then, once more in the same consultation before randomization if they fulfil inclusions criteria and want to participate in the RCT.

**Table 1:** Description of the questionnaires used at baseline, 3 months, 6 months, and 12 months follow-up

Questionnaire	Content	Scores
^a^Numeric Pain Rating Scale (NPRS)	Pain intensity	Numeric scale from 0–10. A change ≥ 2 on NPRS indicates meaningful change.
^a^Neck Disability Index (NDI) (Vernon et al. 1991 [27])	Disability due to neck pain.10 items	Each item is scored from 0 – 5, higher score indicating worse function. Maximum score is 50. A change > 5 points or 10 % is clinically meaningful.
^a^Shoulder Pain and Disability Inventory (SPADI) (Williams et al. 1995 [28])	Pain (5-item) Disability (8-item) 13 items	Each item is scored on a numeric rating scale ranging from 0 to 10. Mean value from the combined scores is given in percent (0–100), higher scores indicating more pain and disability. A change ≥10 on SPADI indicates clinical important change.
^a^Örebro Musculoskeletal Pain Questionnaire -short form (ÖMPQ-SF) (Linton et al. 2011 [26])	Risk for future work disability 10 items	Numeric scale from 0–10, from ‘no pain’ to ‘pain as bad as it could be’ or ‘completely disagree’ to ‘completely agree’. Three items are reversed. The items are being summarized. The total score ranges from 1 to 100 where higher scores indicate higher estimated risk for future work disability.
Norwegian Function Assessment Scale (NFAS) (Brage et al. 2004 [31]; Osteras et al. 2007 [32])	39 items in seven domains: walking/standing, holding/picking up something, lifting/carrying, sitting, coping/managing, cooperation/communication, and senses.	Scored on a 4 point Likert scale, ranging from ‘no difficulty’ to ‘could not do it’, and an average score is calculated.
Subjective Health Complaints inventory (SHC)	Experienced somatic or psychological complaints 29 items	Scored on a 4 point Likert scale, ranging from 0 (no complaints) to 3 (severe complaints). Sumscores are calculated, ranging from 0–87.
Hopkins Symptoms Checklist (HSCL-25) (Derogatis et al. 1974 [34])	Anxiety symptoms (10 items) and Depression symptoms (15 items) 25 items	Scores range from 1 to 4, with 4 indicating severe symptoms. Mean score is reported to 1.23 (95 % CI 1.19–1.30) in a normal population, and cut-off is 1.67 for men and 1.75 for women.
Tampa Scale of Kinesiophobia (TSK) (Kori et al. 1990 [35])	Concerning fear of movement/re-injury 13 items	Scored on a 4 point Likert scale, ranging from 1 (‘strongly disagree’) to 4 (‘strongly agree’). The total score range from 13 to 52. Higher scores indicate higher kinesiophobia
Short Form-12 (SF-12)	Physical and mental health-related quality of life 12-items	Scores ranging from 0 to100. Higher scores reflect better perceived health, with 50 as mean (SD 10) scores for mental and physical dimensions for a healthy population.
Bergen Insomnia Scale (BIS) (Pallesen et al. 2008 [41])	Sleep disturbance 6 items	0–7 days each week. Scored on a 7-point scale; higher scores indicate more severe sleep problems. The total score has a continuous scale (max 42) and normative data has a mean of 10.67 (SD 9.73).

^a^Marked questionnaires are used in the inclusion criteria

**Table 2:** Description of the physical tests performed at baseline and 6 month follow-up

Physical tests	Content	Scores
^a^Global Body Examination – Flexibility (GBE) (Kvåle et al. 2012 [43])	Six tests: truncal flexibility and ability to relax during passive movements: Elbow-drop flexibility, lumbar-sacral flexibility, head rotation resistance and resistance to hip circumduction, hip-knee flexion and arm/shoulder flexion	Each test: 0–7. Total score for Flexibility: 0–42, higher score indicating reduced flexibility. Healthy (*n* = 34): Median = 5.5, mean = 7.2.
^a^ACR-tender points (Wolfe et al. 1990 [53])	18 defined fibromyalgia tender points with four kilos pressure are tested.	Painful points are counted
Back Performance Scale (BPS) (Magnussen et al. 2004 [45]; Myklebust et al. 2007 [46])	Five tests reflecting mobility-related activities for trunk and lower extremities (sock-test, pick-up test, roll-up test, fingertip-to-floor and a lift test where a box weighing 4 kg (women) or 5 kg (men) is lifted from floor to waist for 1 min).	Each test: 0–3. Total score: 0–15 with higher scores indicating worse function. Normative data for people without back pain (*n* = 150): Median = 0, mean = 0.8.
High lift test	The high lift test is a modified lift test included in BPS. The participants lift a box of 2 kg (for women) or 3 kg (for men) from waist to shoulder height and back again.	Number of lifts performed in 1 min, are counted.
Biering–Sørensen test (Biering-Sorensen 1984; [47] Keller et al. 2001 [49])	Static endurance of the back. Participants are positioned prone with the upper body extending beyond the edge of the plinth and the lower body is fixed to the bench with three straps.	Seconds keeping the upper body straight are recorded. Max time 240 s. Healthy (*n* = 31): Median = 138 s
Abdominal endurance/strength (Oja et al. 1995 [51])	Three levels of dynamic sit-up test with increased demand for each level. The participants are supine with the knees flexed and with feet supported on the plinth by the tester.	Completed repetitions are counted (0–15).

^a^Marked tests are used in the inclusion criteria

### Eligibility criteria

#### Inclusion criteria

To fulfil the inclusion criteria the participants must fulfil two of four criteria from the questionnaires, and one of two from the physical tests.

#### Questionnaires

- Pain intensity ≥ 3 on the Numeric Pain Rating Scale (NPRS)
- Sumscore > 30 measured with Örebro Musculoskeletal Pain Questionnaire short form (ÖMPQ-SF)
- Neck Disability Index (NDI) > 14
- Shoulder Pain and Disability Inventory (SPADI) > 20

#### Physical tests

- ≥ 6 tender points according to the American College of Rheumatology criteria for Fibromyalgia
- Sumscore ≥ 7 from five movement tests from the Global Physiotherapy Examination (GPE-Flexibility)

#### Exclusion criteria

- Sick-listed more than 6 months continuously

### Intervention

#### Norwegian Psychomotor Physiotherapy (NPMP) (A)

NPMP was developed in the late 1940s as a result of the collaboration between the physiotherapist Aadel Bülow-Hansen (1906–2001) and the psychiatrist Trygve Braatøy (1904–1953). The treatment approach is based on the premise that the whole body reacts to physical and psychological strain. Over time this may affect flexibility in the body, ability to relax, muscle tension, respiration, posture and body awareness. These elements will also interact [8].

The patient’s history and social situation, as well as the body examination of posture, respiration pattern, movements, muscle quality, autonomic reactions and body awareness, form the basis for the treatment process. The treatment is process-oriented and focusing on the whole patient. The aim is to readjust the posture and the muscle tension by means of breath-releasing massage, touch and movements adapted closely to the patient’s reaction. Movements include grounding (body awareness training in standing and walking), balancing, stretching and relaxation. To increase the patient’s sensation of muscle tension and function, verbal reflections on body experiences is also emphasised and addressed during treatment [8] (Table 3). Consequently, NPMP is not a standardized treatment but rather a continual pragmatic treatment, adjusted to the patient’s bodily experiences and reactions [8]. Each session last for 45–60 min, and is usually received once a week, sometimes every second week.

**Table 3:** Fundamental principles of the two interventions

Norwegian Psychomotor Physiotherapy (NPMP)	Cognitive Patient Education and active individual Physiotherapy (COPE-PT)
NPMP is specialization at post-graduate master level for physiotherapists	COPE is taught to PTs during a 3 days course
Key elements in NPMP	The education program has three basic elements
• Readjust posture	• Reduction of what the patients
• Harmonize muscle tension	• perceive as threatening inputs to the brain
• Harmonize breathing	• Targeting the patients’ own understanding of the pain
• Harmonize movements	• Exposure to the threatening inputs
• Body awareness	
• Each treatment session last 45–60 min	• Each education session last 30 min
• Once a week or every second week	• Education once a week for 4 times
• For 3–6 months	• Followed by active, individualized physiotherapy according to pain problems once a week or every second week
	• For 3–6 months

#### Cognitive Patient Education and active physiotherapy (COPE-PT) (B)

COPE is an “intensive neurophysiology education” program developed by a group of British and Australian researchers as an education program for patients with low back pain. The theory behind the program is primarily based on the neurophysiology of pain, reflected by sensitization and neuronal response to inactivity and movement control [21]. Accordingly, the cognitive elements of the educational program consist of an understanding of pain that differs from the traditional “injury model”, and instead focuses on learning the patient how to better cope with pain and fear of movement.

Studies have documented an additional effect of COPE when combined with physiotherapy [22–24]. In the present study the educational package will be adjusted to patients with extensive musculoskeletal pain or patients with neck and shoulder pain (Table 3). The patients will receive one session weekly with COPE, given by a physiotherapist, maximum four times, followed by active, individualized physiotherapy (PT), as needed and wanted by the patient. The active part of the intervention, usually consist of supervised exercises, and details will be recorded by the PTs.

COPE-PT is thus more an educational package given in a cognitive enhancing manner, followed by active exercise therapy, in contrast to the more process-oriented, body awareness approach found in NMPM, given through massage, relaxation and breathing techniques and exercise, as well as in the dialogue between therapist and patient. As the treatment approaches are pragmatic, number of sessions and content may vary, and the different therapists will register this throughout. Further details can be found in the Additional file 1.

### Baseline and outcome measures

Different measurements will be used at baseline and follow-up in order to detect potential changes both in pain, physical function, mental health condition and quality of life (Tables 1 and 2).

### Primary outcome

The primary outcome is pain, assessed by the *Numeric Pain Rating Scale (NPRS)* which assesses average pain intensity the last 2 weeks on a scale ranging from 0 (no pain) to 10 (worst). The scale has shown better reliability and responsiveness than the visual analogue scale [25].

### Secondary outcomes

The secondary outcomes include additional questionnaires and several physical tests:

#### *Questionnaires* (Table 1)

The *Örebro Musculoskeletal Pain Questionnaire short form (ÖMPQ-SF)* has shown to predict the risk for future work disability. The short form with 10 items is appropriate for clinical and research purposes, and is nearly as accurate as the longer version [26].

The *Neck Disability Index (NDI)*, measures disability due to neck pain, and is a modification of the Oswestry Disability Index [27]. Good reliability and validity of NDI have been demonstrated [27].

The *Shoulder Pain and Disability Inventory (SPADI)* [28], is developed to measure current shoulder pain and function. SPADI has shown to have good reliability, good construct validity [29], to be responsive to change over time in a variety of patient populations, and is able to discriminate adequately between patients with improving and deteriorating conditions [29, 30].

The *Norwegian Function Assessment Scale (NFAS)*, is questioning work-related functioning with basis in the ICF’s classification system [31]. Test-retest reliability has been tested in a normal population and found acceptable [32].

*Subjective Health Complaints Inventory (SHC)* consists of items regarding subjective somatic or psychological complaints experienced during the last month. The SHC inventory has shown satisfactory test-retest reliability in students and patients with LBP [33].

The *Hopkins Symptoms Checklist (HSCL-25)* measures anxiety and depression symptoms. The HSCL has been shown to have satisfactory validity and reliability in psychiatric outpatients and in a normal population [34].

The *Tampa Scale of Kinesiophobia (TSK)* [35] in short form measures fear of movement and re-injury. The TSK has been validated in numerous studies including patients with neck pain, acute and chronic LBP and fibromyalgia [35–38].

The *Short Form-12 (SF-12)*, which is a 12-item version of the SF-36, measure physical and mental health-related quality of life [39]. The SF-12 has shown good internal consistency, validity, and responsiveness in patients with LBP [40].

The *Bergen Insomnia Scale (BIS)* measures sleep disturbance. BIS can refer to high internal consistency, adequate reliability and good convergent and discriminative validity [41].

Sick leave will be measured by number of self-reported days of absence from work.

At 3 and 6 months the patients in both arms of the RCT will also fill in the *Client Satisfaction Questionnaire (CSQ-8)* [42].

#### *Physical tests* (Table 2)

Six tests from the *Global Body Examination (GBE)* assess truncal flexibility and ability to relax during passive movements. Discriminating ability between healthy and different patients groups has shown to be very good to excellent [43]. Good inter-tester reliability has been demonstrated in a former version of the GBE [44].

*Back Performance Scale* (BPS), consists of five tests reflecting mobility-related activities for trunk and lower extremities and include a sock-test, a pick-up test, a roll-up test, a fingertip-to-floor test and a lift test where a box weighing 4 kg (women) or 5 kg (men) is lifted from floor to waist for 1 min) [45, 46].

A *high lift test* is a test quite similar to the lifting test in the Back Performance Scale (BPS).

Static endurance of the back extensors is assessed with *the Biering–Sørensen test* [47]. Test-retest reliability has been reported as satisfactory, but variability has been high [48–50].

Abdominal strength is assessed with a three levels *dynamic sit-up test* with increasing demands for each level [51, 52].

*The American College of Rheumatology* (ACR) of fibromyalgia has defined *18 tender points* [53]. In addition to counting the number of tender points, the examiner gets an impression whether pain is localized or widespread. A pain drawing is also used for this.

Recently an inter-tester reliability study of all the physical test have been performed, showing high to very high reliability of all the tests (ICC2,1 from 0.80 to 0.94) [54].

### Sample size calculation

The aim is to enroll a total of 128 participants in the study. Power calculations are based on a power of 80 % and a significance level of 5 % to detect a meaningful difference between the two intervention groups on the patient-reported primary outcome measure. Based on previous studies using the NPRS to measure pain intensity, a clinically significant difference can be estimated to be approximately 2 points with a standard deviation of 2. Power calculations based on these data indicate that a total of 16 participants in each group will be sufficient to detect a treatment difference after ended intervention. However, long term follow-up (12 months) and expectations of decrease in difference between the two intervention groups with 1 point, there is a need to increase each group to 64 participants.

### Randomization method

Block randomization of the study sample will be employed, using a computer generated list with blocks of six to ensure similar number of participants in both intervention groups. Information about intervention allocation (A or B) is then put into premade and ordered envelopes. The sealed envelopes are kept in a locked box by receptionists who have no information about the content. The receptionist hands out one envelop consecutively to the participants after eligibility and baseline measurements. The envelope contains information regarding treatment and names of treating therapists (A or B), and the participants are invited to call one of them for an appointment.

### Follow-up

All the participants will be followed-up at three different points. At 3 and 12 months questionnaires will be sent to the participants’ home address, and returned by them in a pre-paid envelop. At 6 months the participants will undergo a functional examination and fill inn questionnaires (the same as at baseline) (Table 2 and 3). A receptionist makes the 6 months follow-up appointments, and a blinded physiotherapist, not knowing what treatment the patient has been given, will perform the examination (Fig. 1).

### Statistical analysis

All statistical analyses will be carried out using R version 3.2.5 (The R Foundation for Statistical Computing, www.r-project.org) and SPSS version 24 (SPSS Inc., Chicago, IL, USA) for windows. All tests will be two-sided, and *p* values less than 0.05 will be considered statistically significant.

Continuous variables will be reported as means ± SEM and categorical variables as percentages (%) ± SEM. The chi-square test will be used to test for group difference in percentages of categorical variables, while the two-sample *t*-test will be used to test for group difference in means of continuous variables. Analog non-parametric tests will be evaluated when strongly skewed distributions.

To estimate and compare the treatment effect of the two specific interventions at the four time points (baseline, 3, 6 and 12 months) we will use mixed effects models. All models will define treatment, time and treatment-by-time interaction as fixed effects, whereas a random intercept will be specified to account for correlated observations of the same individual (an exchangeable correlation structure assumed). Depending on the measurement level of the outcome under study, treatment effects will be presented as relative estimates (e.g., odds ratios, relative risk) or predicted mean differences with 95 % confidence intervals, corrected for multiple testing. Age, gender, body mass index, duration of pain and work status will be evaluated as additional adjustment variables.

We cannot exclude the possibility that missing outcome data may occur for a proportion of participants. The mixed effects model will produce unbiased effect estimates as the model uses a likelihood-based estimation procedure, provided the data are missing at random (MAR). Additionally, a multiple imputation method for missing data will be utilized if missing data occur in covariates as well.

Effect size (ES) for mean change will also be calculated by subtracting post-test scores (6 and 12 months) from baseline in the two groups, dividing it by the standard deviation (SD) of the change score.

## Discussion

We have presented the protocol for a pragmatic RCT, investigating the effectiveness of Norwegian Psychomotor Physiotherapy (NPMP) for patients with neck and shoulder pan and/or widespread pain, when compared to cognitive pain education combined with active physiotherapy (COPE-PT). The primary outcome of this study will be pain, assessed by the *Numeric Pain Rating Scale (NPRS).* Wand et al. [55] argue that there is a need of both self-reported and physically tested functioning to better grasp aspects of disability among patients with musculoskeletal pain. Accordingly, the secondary outcomes include both self- reported and tested function.

When choosing outcome measures, it is crucial to ensure congruence between the content of the treatment and the outcome. The NPMP is based on the understanding of the body as an integrated physical and psychological phenomenon and thus bodily change in the treatment might be followed by an emotional change. In order to grasp different aspects of the treatment, several secondary outcome measures of both physical function and mental health are necessary and therefore included in this study. In addition, all measurements are well-known and shown to be valid and reliable, as well as they measure different aspects of functioning according to the International Classification of Functioning and Health [56].

Several potential confounding factors may have an impact on both the primary and secondary outcomes. These may include factors as participant age, duration of pain, and psychological factors. Our randomized design is expected to distribute these variables equally between the two intervention groups. The blinding of the physiotherapist performing and collecting the follow-up data will also enhance the validity of the study. The performance of power analysis and sample size estimation is also an aspect to improve the precision to provide reliable answers to the primary outcome measure.

However, compliance with intervention might be a possible threat to the validity. This is a challenge because different physiotherapists (*n* = 10) are involved in the two treatment approaches. In addition NPMP is individually tailored which further increases the complexity, since the treatment will not only vary between individuals but also between physiotherapists. In order to ensure compliance, all the physiotherapists in both treatment approaches will be regularly followed by separate meetings for therapists in each of the two interventions, discussing how they implement and internalize the treatment during the study period. In addition, as the treatments are pragmatic the therapists will register more details regarding content and number of treatment given. A strength, however, is that the treatment will occur in natural settings, hence, its practicality and feasibility may be high.

In summary, the findings of the present study may give an important contribution to our knowledge of the outcome of NPMP, on patients with long-lasting widespread musculoskeletal pain and/or pain located to the neck and shoulder region.

## Abbreviations

NPMP, norwegian psychomotor physiotherapy; COPE-PT, cognitive pain education and active physiotherapy; RCT, randomized controlled trial; NPRS, numeric pain rating scale; ÖMPQ-SF, Örebro musculoskeletal pain questionnaire short form; NDI, neck disability index; SPADI, shoulder pain and disability inventory; GPE-Flexibility, global physiotherapy examination – flexibility; SHC, subjective health complaints inventory; HSCL-25, Hopkins symptoms checklist; TSK, tampa scale of kinesiophobia; SF-12, short form-12; BIS, bergen insomnia scale; CSQ-8, client satisfaction questionnaire; BPS, back performance scale; ACR, The American College of Rheumatology; ICC, intraclass correlation coefficients; SEM, standard error of measurement; ES, effect size; SD, standard deviation

## Additional file

Additional file 1: Norwegian Psychomotor Physiotherapy (DOCX 19 kb)
