# Supplementary material for: Study protocol for Norwegian Psychomotor Physiotherapy versus Cognitive Patient Education in combination with active individualized physiotherapy in patients with long-lasting musculoskeletal pain – a randomized controlled trial
Source: BMC Musculoskelet Disord. 2016 Aug 5;17:325. doi: 10.1186/s12891-016-1159-8 (PMC4974790; doi:10.1186/s12891-016-1159-8)
Supplement: Additional file 1: — Norwegian Psychomotor Physiotherapy (DOCX 19 kb) [file 12891_2016_1159_MOESM1_ESM.docx]

Norwegian Psychomotor Physiotherapy

The perspective of Norwegian Psychomotor Physiotherapy (NPMP) is that physical, psychological and social strains influence the whole body affecting muscle tension, respiration, posture, balance and flexibility (Thornquist and Bunkan, 1991). The treatment modality was developed in the late 1940s as a result of the collaboration between the physiotherapist Aadel Bülow-Hansen (1906–2001) and the psychiatrist Trygve Braatøy (1904–1953).

The typical NPMP examination includes the patient’s history of complaints and an assessment of bodily functions. The main purpose of the case history is to grasp the patient’s description, experience and understanding of bodily symptoms, and how these are related to past experiences as well as daily life. The extensive body examination includes assessment of posture, respiration, movements, muscle quality, autonomic reactions and perceived body awareness. The patient’s posture and respiration is examined in standing, sitting and lying positions. Assessment of movements includes active functional movements, passive movements and ability to give in to the force of gravity. Muscle quality is examined by palpation in order to evaluate muscular tension. The assessment of respiration is considered most important, as the rhythm of breathing indicate the patients’ resources and potential for change (Thornquist and Bunkan, 1991).

The aim of treatment is to readjust the posture and the muscle tension by means of breath-releasing massage, touch and movements adapted closely to the patient’s reaction. Local symptoms are always assessed in relation to the tension pattern in the whole body. Even if the symptoms are located in the neck it may be considered necessary to focus on tension pattern in the lower limbs, because better balance often is a prerequisite for improvement of the symptoms of the neck. But also tension in the neck may prevent good function of the back and lower limbs. In other words, one part may influence the function in other parts of the body. Thus NPMP always encompasses treatment of the whole body (person). To increase the patient’s sensation of muscle tension and function, verbal reflections on body experiences are emphasised and addressed during treatment. Awareness of own bodily reactions, like patterns of tension and movements is, accordingly, considered an important prerequisite for functional change, which may also indicate an emotional change (Thornquist and Bunkan, 1991).

Cognitive Patient Education

The "intensive neurophysiology education" program has been developed by a group of British and Australian researchers as an education program for patients with LBP. The cognitive elements of this program consist basically of an understanding of pain that differs somewhat from the traditional "injury model". This theory is primarily based on the neurophysiology of pain, reflected by sensitization and neuronal response to inactivity and movement control (Butler and Moseley, 2003). Based on this, the education program has three basic elements

- Reduction of what the patients perceive as threatening inputs to the brain
- Targeting the patients' own understanding of the pain
- Exposure to the threatening inputs

The intervention is usually given as four 30-minute sessions once a week. Each session has a specific content of education and discussion in a one-to-one setting between the physiotherapist and the patient. Initially the discussion concerns thoughts and fears the patients might have according to their pain. Then the education program deals more with exposure to movements and daily activities that the patients more or less avoid because of fear of provoking pain. As homework between the sessions, the patient is asked to identify barriers to normal functioning, reasons for fear avoidance behavior and other reflections related to the education. They are also asked to make specific registrations regarding function, pain and work absence. The cognitive based education program is specified in a manual with a written summary of the content of each session.

In addition to the cognitive based education program the patients will receive active physiotherapy consisting of whatever is the usual procedure for the individual physiotherapist according to the patient’s pain history and problems.
